# Monitoring protease activity in biological tissues using antibody prodrugs as sensing probes

**DOI:** 10.1038/s41598-020-62339-7

**Published:** 2020-04-03

**Authors:** Olga Vasiljeva, Elizabeth Menendez, Margaret Nguyen, Charles S. Craik, W. Michael Kavanaugh

**Affiliations:** 1 CytomX Therapeutics, Inc.; 151 Oyster Point Blvd. Suite 400, South San Francisco, California, 94080 USA; 2 Department of Pharmaceutical Chemistry, University of California, 600 16th Street, San Francisco, CA, 94143 USA

**Keywords:** Biochemistry, Proteases, Cancer microenvironment, Cancer microenvironment, Cancer models, Biological techniques, Sensors and probes, Fluorescent proteins

## Abstract

Proteases have been implicated in the development of many pathological conditions, including cancer. Detection of protease activity in diseased tissues could therefore be useful for diagnosis, prognosis, and the development of novel therapeutic approaches. Due to tight post-translational regulation, determination of the expression level of proteases alone may not be indicative of protease activities, and new methods for measuring protease activity in biological samples such as tumor biopsies are needed. Here we report a novel zymography-based technique, called the IHZ^TM^ assay, for the detection of specific protease activities *in situ*. The IHZ assay involves imaging the binding of a protease-activated monoclonal antibody prodrug, called a Probody^®^ therapeutic, to tissue. Probody therapeutics are fully recombinant, masked antibodies that can only bind target antigen after removal of the mask by a selected protease. A fluorescently labeled Probody molecule is incubated with a biological tissue, thereby enabling its activation by tissue endogenous proteases. Protease activity is measured by imaging the activated Probody molecule binding to antigen present in the sample. The method was evaluated in xenograft tumor samples using protease specific substrates and inhibitors, and the measurements correlated with efficacy of the respective Probody therapeutics. Using this technique, a diverse profile of MMP and serine protease activities was characterized in breast cancer patient tumor samples. The IHZ assay represents a new type of *in situ* zymography technique that can be used for the screening of disease-associated proteases in patient samples from multiple pathological conditions.

## Introduction

Proteases catalyze the hydrolytic cleavage of peptide bonds and can be divided into five distinct classes based on their catalytic mechanisms: serine, cysteine, aspartic, metallo- and threonine proteases^1^. Proteases are involved in numerous important normal physiological processes including protein turnover, nutrient digestion, fertilization, cell differentiation and growth, the immune response, and apoptosis. The activity of proteases is normally tightly controlled through multiple redundant mechanisms, including regulation of biosynthesis, activation of inactive precursors known as pro-enzymes or zymogens, and by the binding of endogenous inhibitors and cofactors^2^. Inappropriate proteolysis can have a major role in development of pathological conditions such as cancer and cardiovascular, inflammatory, neurodegenerative, bacterial, viral, and parasitic diseases. Multiple proteases have been associated with cancer; among them, metalloproteinases, serine and cysteine proteases have taken on heightened importance due to their significant up-regulation in the cancer microenvironment and execution of diverse functions at different stages of malignant progression, including tumor angiogenesis, invasion, and metastasis^3^. Due to their involvement in multiple pathological processes, proteases represent attractive biomarkers or drug targets in a number of therapeutic areas^4^.

Most available techniques for identifying and characterizing proteases in tissues are focused on the detection of mRNA or protein expression and do not provide information on protease activity. This is an important limitation because proteases can be expressed at high levels in their inactive form or  present in complex with endogenous inhibitors^5^. Traditional zymography enables detection of functional proteases by use of reagents that visualize substrate degradation; however, conventional zymography techniques have significant limitations^6^. For example, *in gel* zymography uses tissue homogenates, which precludes localization of enzyme activity within tissue and may be difficult to interpret, because of the inappropriate interaction of proteases or their inhibitors in the homogenate that have may have been previously separated in distinct compartments of the intact cells or tissues^7^. Techniques such as *in situ* zymography use cryo-preserved or fresh tissues rather than homogenates^8^, but identification of the active proteases in the tissue relies on the specificity of the substrates used. Commonly used reagents for *in situ* zymography, such as DQ-collagen or DQ-casein, are based on substrates that can be cleaved by many different proteases, making interpretation of the protease biology difficult. Moreover, whereas the activity-based probe techniques that use more selective protease substrates could be more precise in cataloging specific protease activity, they have limited ability to identify the localization and mapping of high protease activity sites in tissues^9,10^. Recently, several active site-specific anti-protease antibodies were developed that can detect the active form of proteases by immunohistochemical methods; however, this approach is limited to small subset of proteases^11–13^.

Given the potential broad utility of protease activity assessment, we have developed a technology, which can be applied to profiling and monitoring protease activity in any biological tissue, that is based on the unique features of a protease-activated antibody prodrug. This technology enables detection of active proteases and can predict efficacy in tumor models in animals. Furthermore, we demonstrated that our technology can be used for assessment of protease activity in cancer patient tumor samples, providing the potential for new predictive biomarkers.

## Results

### IHZ^TM^ technology measurement of protease activity *in situ*

**Figure 1:**
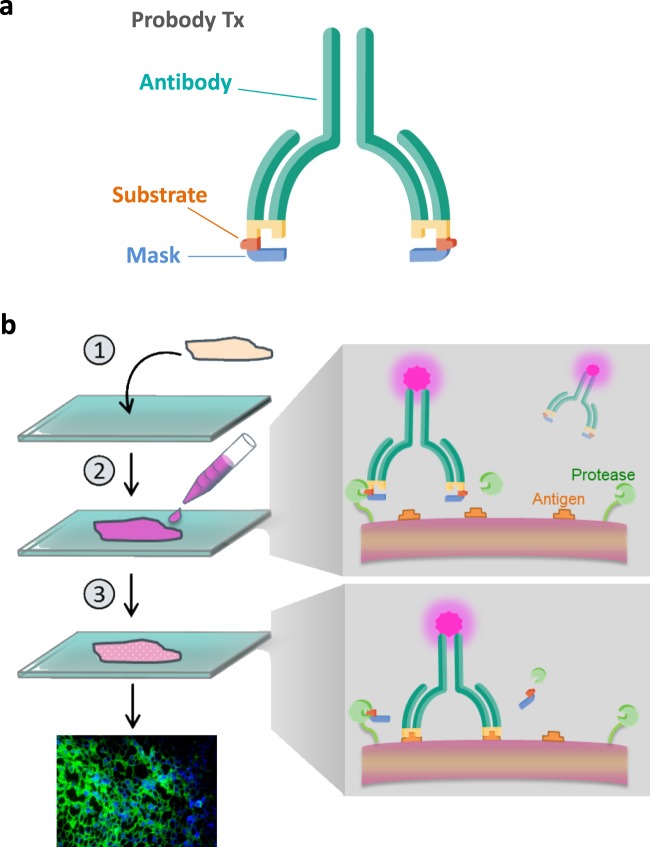
Overview of a Probody construct and IHZ technology. (**a**) A Probody construct contains a masking peptide (blue) that blocks the antigen-binding site (yellow) and is linked to the antibody (green) through a protease substrate-containing linker (orange). (**b**) Schematic overview of IHZ assay. (1) A cryopreserved tissue section is mounted on a slide; (2) A labeled Probody construct is incubated with the tissue; (3) In the presence of protease activity, activation of Probody construct occurs, resulting in dissociation of the masking peptide and binding of the unmasked antibody to the tissue antigen. After extensive washing, binding of the un-masked antibody to the tissue section is detected by immunohistochemical staining techniques.

To enable specific detection of protease activity in biological tissues, a new approach based on a protease-activatable antibody prodrug was developed. Upon its activation by tissue endogenous proteases through specific substrate hydrolysis, the released antibody would bind to the tissue antigen, thus serving as a biomarker of specificity of the protease. In this work, antibody prodrugs engineered to bind target antigen after activation by a protease, called Probody^®^ therapeutics, are used as the assay reagents. A Probody construct is comprised of the monoclonal antibody and a masking peptide that physically blocks the antigen-binding site of the antibody. The mask is identified by screening a peptide library^14^ and is fused to the amino terminus of the antibody light chain through a linker containing a substrate that is cleavable by selected protease class(es) of interest (Fig. 1a). Protease-specific substrates are identified by screening a peptide library using a tagged bacterial display system that enables positive and negative selection by fluorescence-activated cell sorting (FACS) of peptides cleaved by recombinant proteases of interest^15^. When the Probody therapeutic comes in contact with the appropriate active protease, the mask is removed, and the released antibody becomes competent to bind antigen.

The IHZ technique involves incubation of a Probody construct with a cryopreserved tissue section. If the tissue section includes proteases that activate the Probody construct, binding of the released antibody to antigen present in the sample can be detected by immunofluorescence (IF) or chromogenic staining (Fig. 1b). By generating Probody constructs with differing protease specificities, the profile of specific protease activities in the tissue section can be characterized. Probody constructs can be directed against any antigen that is sufficiently abundant in the samples of interest for detection using immunofluorescent techniques. Such an antigen could be either specific for the tissue under study or widely expressed to enable binding to a broad spectrum of different tissues. Furthermore, to account for the possible differences in antigen density in individual tissue sections, normalization of IHZ signal to the target expression can be applied.

**Figure 2:**
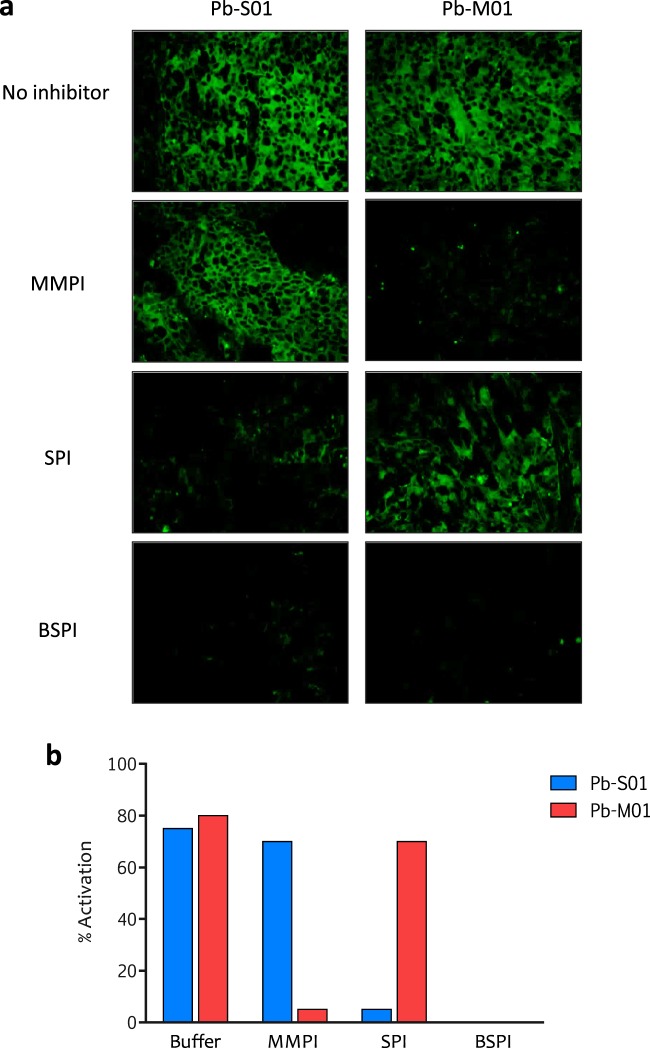
Validation of the IHZ assay for different protease specificities in xenograft tumor tissue. (**a**) The EGFR IHZ assay was performed with anti-EGFR Probody constructs containing the serine protease substrate LSGRSDNH (Pb-S01) and the MMP substrate PLGL (Pb-M01) on 5 µm cryopreserved sections of H292 lung xenograft tumor tissue. Specific protease inhibitor cocktails were used to demonstrate selectivity of Probody molecule activation. (**b**) Quantitative assessment of the IHZ fluorescent signal for both Probody constructs in the presence and absence of protease inhibitors, as described in Methods. MMPI, Matrix Metalloproteinase Inhibitor GM6001; SPI, serine protease inhibitor cocktail; BSPI, broad spectrum protease inhibitor cocktail.

Preclinical proof of concept of the Probody platform was demonstrated using an anti-EGFR Probody therapeutic based on a cetuximab derivative (C225)^16^. To demonstrate the ability of the IHZ assay to assess activity of proteases with different specificities, we engineered two different anti-EGFR Probody constructs with a previously described mask^16^. The first construct contained a substrate (LSGRSDNH) specifically cleavable by at least two serine proteases: matriptase (MT-SP1), a tumor-associated type II transmembrane serine protease, and urokinase-type plasminogen activator (uPA). The second construct contained a substrate (PLGL)^17^ that is cleavable by several matrix metalloproteinases (MMPs). These Probody constructs are referred to as Probody construct Pb-S01 (Probody-S01) and Probody construct Pb-M01 (Probody-M01), respectively. We initially evaluated H292 human non-small cell lung cancer xenografts, which are characterized by high EGFR expression and *in vivo* response to cetuximab^15^. As shown in Fig. 2, Pb-S01 and Pb-M01 both demonstrated EGFR staining in H292 xenograft tumor sections. The staining with both Probody reagents was almost completely inhibited by the pretreatment of the tissues with a broad-spectrum protease inhibitor (BSPI) cocktail. Further, most of the IHZ signal from Pb-S01 was inhibited by serine protease-specific inhibitors, whereas MMP-specific inhibitors had almost no effect (Fig. 2). Similarly, staining of Pb-M01 was abolished by pretreatment of the tissue section with MMP-specific inhibitors, while serine protease inhibitors had minimal effect. These data demonstrate that staining is dependent on protease activity in the tissue section and corroborate the specificity of the selected substrates and of the assay. Quantification of the intensity of staining of the Probody construct relative to the parental antibody was normalized for EGFR expression determined by co-staining with noncompeting EGFR antibodies, as described in Methods and depicted in Fig. 2b. Furthermore, this relative quantification approach was shown to be reproducible across independent experiments (Supplementary Fig. 1).

### IHZ technology enables comparison of protease activity between xenograft tumor models

We next investigated if the IHZ assay approach can enable profiling and comparison of protease activity between different tumor models. To address this question, two xenograft tumor models expressing EGFR were selected for characterization of matriptase and uPA protease activity: (1) H292, derived from a mucoepidermoid lung carcinoma; and (2) FaDu, derived from a head and neck carcinoma. To differentiate between matriptase and uPA proteolytic activities in these xenograft tumor models we have used Probody probes directed against EGFR and containing a substrate cleavable by both matriptase and uPA (Pb-S01), or Probody construct Pb-S02 (Probody-S02) containing a uPA selective substrate (TGRGPSWV)^18^.

**Figure 3:**
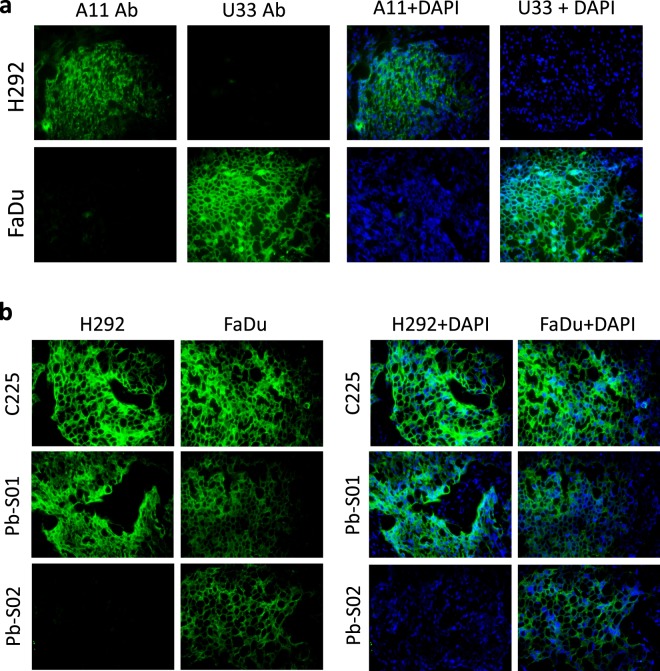
Assessment of uPA and matriptase activity in H292 and FaDu xenograft model tissue sections. (**a**) The presence of active matriptase and uPA in tissues was confirmed by the use of active site-specific antibodies, A11 and U33, respectively. H292 lung cancer xenograft tumors contained active matriptase (A11 staining), but not active uPA (U33 staining). FaDu head and neck cancer xenograft tumors contained active uPA (U33 staining) but not active matriptase (A11 staining). (**b**) H292 and FaDu xenograft tumor tissue sections were incubated with C225 antibody, the Probody reagent Pb-S01 containing a linker cleavable by both matriptase and uPA, and the Probody reagent Pb-S02 containing a linker cleavable by uPA but not by matriptase. Pb-S01 activation and binding was detected in both xenograft tissues, whereas Pb-S02 binding was detected in FaDu xenograft tumors only. Parental antibody C225 binding confirmed the presence of approximately equal levels of EGFR receptor in both xenograft tissues. Original magnification ×40.

To independently establish the protease activity profile of these tumors, we used antibodies that specifically bind to the active-site of matriptase, called A11^12^, or uPA, called U33^19^. Both of these antibody reagents bind only to the active confirmation of the enzyme, but not to its zymogen form or to its complex with endogenous inhibitors, and therefore serve to establish the presence of active protease activity in samples independently from the IHZ assay. Protease activity was assessed in H292 and FaDu tumors by IF staining with these antibodies of the corresponding tumor tissue sections (Fig. 3a). These cell line xenograft models were chosen because matriptase and uPA are commonly upregulated in human cancer^3^. H292 xenograft tumors stained positively for active matriptase and negatively for active uPA (Fig. 3a, upper row), whereas FaDu tumors had the opposite protease activity profile characterized by the presence of uPA activity and absence of matriptase activity (Fig. 3a, lower row). Next, both xenograft tumor tissues were profiled by IHZ assay with Pb-S01 and Pb-S02 Probody reagents directed against EGFR. Although both tumors were characterized by a similar level of EGFR expression (Fig. 3b, upper row), Pb-S02 IHZ signal was detected only in the FaDu xenograft and not in the H292 tumor tissue (Fig. 3b). To confirm the cleavability of Pb-S02 by uPA under the IHZ assay conditions in H292 tumor samples, Pb-S02 IHZ assay of H292 tumors was repeated with the addition of recombinant uPA to the assay reaction. This resulted in strong staining similar to the parental antibody (cetuximab, C225) (Supplementary Fig. 2). Further, Pb-S01, which can be activated by both matriptase and uPA proteases, demonstrated IHZ signal in both H292 and FaDu tumor tissues (Fig. 3b). The attribution of IHZ assay signal for Pb-S01 in H292 tumor to matriptase was confirmed by pretreatment of tissue with A11 active-site antibody, a specific matriptase inhibitor, that resulted in ablation of the IHZ signal (Supplementary Fig. 3). Taken together, these results demonstrate a correlation of the IHZ assay with the specific protease activity as measured by site-specific anti-protease antibodies. Further, the data demonstrate the IHZ assay can detect different protease activities in xenograft tumor samples.

**Figure 4:**
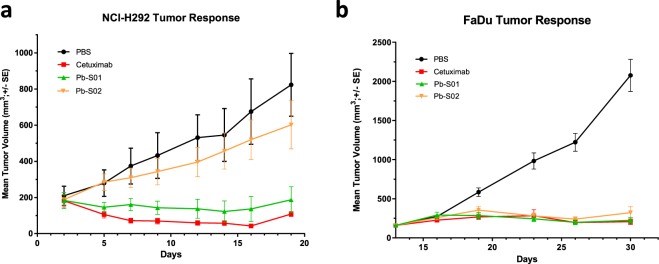
Probody constructs Pb-S01 and Pb-S02 demonstrate differential efficacy in H292 and FaDu xenograft mouse models. (**a**) H292 xenograft tumor–bearing mice (n = 8 per treatment group) were treated intravenously once weekly (25 mg/kg) for 4 weeks with cetuximab (red), Pb-S01 (green), Pb-S02 (yellow) or vehicle (10 ml/kg; black). Data are presented as mean tumor volume ± SEM. (**b**) FaDu xenograft tumor–bearing mice (n = 8 per treatment group) were treated intravenously by two weekly doses (25 mg/kg, blue) of cetuximab (red), Pb-S01 (green), Pb-S02 (yellow) or vehicle (10 ml/kg; black). Data are presented as mean tumor volume ± SEM (n = 8 per treatment group).

We next investigated whether the IHZ assay correlated with antitumor efficacy of the EGFR-directed Probody reagents *in vivo* in both xenograft tumors (Fig. 4a,b). Consistent with the absence of uPA activity in H292 tumors capable of removing the mask and activating Pb-S02, no effect on tumor growth was detected after treatment with Pb-S02 in the H292 xenograft tumor model (P = 0.33). However, Pb-S02 had equipotent efficacy to the unmasked parental antibody cetuximab in the FaDu xenograft model, where uPA activity was detected (80%, P < 0.0001), thus corroborating IHZ assay findings. Moreover, as would be expected from the active site antibody IF staining and IHZ assay data reported above, Pb-01 was equally efficacious in both xenograft models (Fig. 4a,b). These data demonstrate a correlation of the specific protease activity as measured by the IHZ assay with the antitumor efficacy of the Probody constructs in xenograft models.

### Assessment of protease activity in patient -derived tumor samples

**Figure 5:**
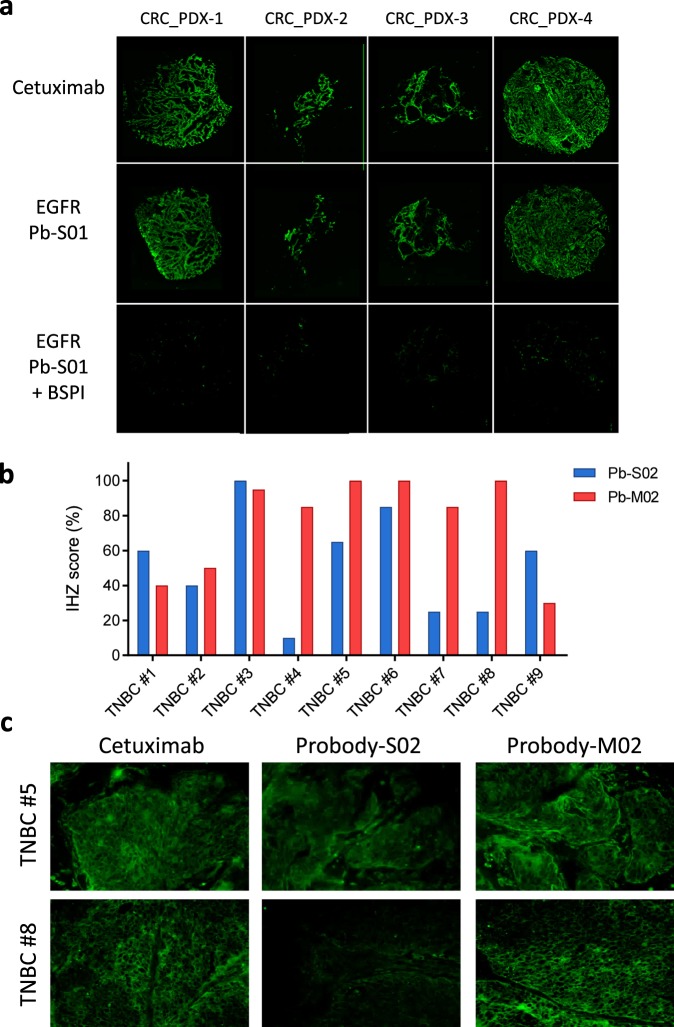
Protease activity in PDX and patient tumor tissues as determined by the IHZ assay. (**a**) Pb-S01 activation in representative CRC PDX tumors (second row images). Staining with parental antibody cetuximab to EGFR was used for normalization (first row images) and BSPI was added to the IHZ assay for quantification of protease-dependent staining (third row images). (**b**) Characterization of uPA and MMP-14 protease activity in tumor tissues of triple negative breast cancer (TNBC) patients by Pb-S02 (blue) and Pb-M02 (red), respectively. IHZ staining reflecting protease activity was quantified as described in Methods. (**c**) Representative images of TNBC patient samples stained with Pb-S02 and Pb-M02 by the IHZ assay and with cetuximab as a control.

To expand the application of IHZ technology, we first used Pb-S01 to profile protease activity in colorectal cancer (CRC) patient-derived xenograft (PDX) tumor samples. A frozen tissue microarray (TMA) was constructed from 15 human CRC tumors that were derived from primary tumors or distant metastases engrafted directly into nude mice without intervening growth in cell culture^20^. IHZ screening of the TMA with Pb-S01 demonstrated staining similar to that of parental antibody cetuximab, which was abolished by the tissue pretreatment with BSPI (Fig. 5a). The calculated Pb-S01 IHZ assay scores ranged from 65% to 100% (Supplementary Table 1), thus providing evidence for the presence of matriptase and/or uPA activity in all of the PDX tumors tested.

Next, to evaluate the applicability of the IHZ assay approach for characterization of protease activity in human tissues such as tumor biopsies, we have assessed protease activity of different patient tumor samples of one indication. Nine triple-negative breast cancer (TNBC) patient tumor samples were stained with Probody constructs containing either a selective uPA substrate (Pb-S02) or an MMP-14 substrate (SLAPLGLQRR)^21^, termed Pb-M02 (Supplementary Table 2). All patient tissue samples showed some staining with both Probody constructs; however, the relative amount of Probody molecule activation by uPA and MMP-14 differed among the patients (Fig. 5b,c). These data demonstrate the utility of IHZ technology for evaluation and comparison of activity of specific proteases in variety of tissues, including clinical patient samples. This technology could also be used to profile human tissues for spatial information on protease activity that could support the development and optimization of other protease-sensitive technologies and therapeutics.

## Discussion

In the present study, we have demonstrated a novel *in situ* zymography approach that allows detection of specific proteolytic activity in biological samples. After exposure of tissue to a protease-activatable antibody prodrug and detection of antigen binding using immunofluorescence staining, endogenous protease activity can be assessed. Using this method, we were able to characterize and compare the activity of proteases of different classes in various tissue samples. As compared to the currently available methods for detection of protease activity, the IHZ assay has several advantages. First, it allows direct visualization and rapid assessment of a variety of proteases from different families and classes by applying Probody constructs containing protease-specific substrate sequences. Second, this assay requires only a small amount of tissue by virtue of immunofluorescent microscopy-based detection. This feature is of particular utility when analyzing small clinical samples, such as those obtained by fine needle biopsy. The fact that the IHZ assay relies on the well-established robustness of conventional immunohistochemical detection techniques is also an important strength of this assay, and more sophisticated imaging technologies and software algorithms for fluorescent image analysis and quantification could improve the accuracy and precision of this zymography technology.

The ability to detect proteolytic activity of individual proteases is important when assessing the role of proteases in physiological or pathological conditions. It is known that proteases can be present in an inactive zymogen form or can be inhibited by inhibitors present in circulation and tissues^2^. Therefore, the ability to measure activity of proteases rather than their concentration provides unique and important information about their potential biological function. Furthermore, the target binding-based read-out of this assay could allow one to simultaneously characterize, on a single tissue section, proteolytic activity of the tissue and expression of the antigen to which the Probody construct is directed. The described method can be adapted to virtually all proteases, provided that selective substrates and the optimal conditions for individual proteases are available and applied. In cases where substrates are not sufficiently selective due to overlapping protease specificities, the IHZ assay can be complemented with the use of selective inhibitors or their combination. In addition, similar to other zymography-based techniques, the IHZ assay is time dependent and therefore can be optimized accordingly to any given protease, substrate, or tissue type to provide the optimal window for detection and protease activity ranking. Among the methods that could be used to increase the sensitivity of the IHZ assay, we believe that the use of fluorescent polymerization-based signal amplification^22^ or carbon nanotube-enhanced polarization of fluorescent peptides^23^ may be useful to increase sensitivity of the method for low expression targets.

Overall, given a growing body of evidence highlighting the importance of proteases in numerous biological processes, the new information gained from the IHZ assay may further the understanding of the function of proteases in normal and diseased tissues and inform the design of novel diagnostic and therapeutic strategies for multiple diseases, including cancer.

## Methods

### EGFR Probody construct expression, purification and labeling

Cetuximab-based Probody constructs were engineered, expressed, and purified as described by Desnoyers *et al*.^16^. In brief, Probody constructs were cloned into a modified pcDNA3.1 mammalian expression vector (Life Technologies), expressed in Expi293 cells (Life Technologies), and affinity purified using MabSelect Sure protein A columns (GE Healthcare). The purity of the Probody constructs was analyzed by SDS-PAGE under reducing and non-reducing conditions, and homogeneity was analyzed by size-exclusion chromatography using a Superdex 200, 10/300 GL column (GE Healthcare). Probody monomers (>95%) with a purity of more than 95% were obtained.

Antibodies and Probody constructs were conjugated with a near-infrared fluorescent Alexa Fluor 680 or 750 dye (AF680/750, ThermoFisher Scientific) using N-hydroxysuccinimide (NHS) ester reaction. Constructs and amine-reactive fluorescent dyes were incubated for 1 hour at room temperature and the reaction was stopped with 1 M Tris-HCl buffer, pH 8.5. To separate labeled antibody and Probody therapeutics from free dye, Zeba desalting columns (Life Technologies, 87768) were utilized. Antibodies and Probody therapeutics with degree of labeling (DOL) of 2 to 3, as determined by NanoDrop spectrophotometer (ThermoFisher Scientific), were used in the imaging studies.

### EGFR IHZ assay protocol

H292 and FaDu xenograft tumor samples were flash frozen in liquid nitrogen and embedded in OCT. CRC PDX tumor samples were purchased from Oncodesign (Dijon, France). Human tissue samples were provided by ProteoGenex, Inc. (Inglewood, CA). All specimens are collected under ethical regulations and in accordance all applicable (local and international) laws. No ethics committee approval is required for work with archival human samples obtained from commercial tissue bank (ProteoGenex, Inc.). The tissue samples were frozen with either liquid nitrogen or dry ice within 1 hour of surgical removal. Five µm frozen tissue sections were cut on a cryostat (Microm HM550, ThermoFisher) and placed on glass slides. Slides were kept at −20 °C until ready for use or stored at −80 °C for long-term storage. Slides were brought to room temperature and allowed to dry for 30 minutes before beginning the procedure. A hydrophobic barrier was drawn around the tissue section (ImmEdge Hydrophobic Barrier Pen, Vector Laboratories). The slides were then rinsed in phosphate-buffered saline (PBS) and incubated with either 100 µL 50 mM Tris-HCl buffer pH 7.4, containing 150 mM NaCl, 100 µM ZnCl2, 5 mM CaCl2, and 0.05% Tween 20 (Tris Buffer) or 100 µL of 1:100 dilution of broad spectrum inhibitor cocktail set III (539134, EMD Millipore, Billerica, MA) and 50 mM EDTA in Tris Buffer (BSPI). In some experiments, a serine protease-specific inhibitor cocktail set I (Calbiochem) or the MMP-specific inhibitor GM6001 (Galardin; Calbiochem) was used in place of BSPI. The slides were then incubated for 30 minutes at room temperature. The samples were stained by adding 100 µL of a solution containing 1 µg/mL AF680-labeled Probody construct or cetuximab in Tris Buffer, and 1:50 dilution of a different anti-EGFR antibody (Cell Signaling) to enable normalization of the IHZ signal to EGFR receptor density, then incubated for 1 hour at room temperature. The tissue was then washed in three changes of PBS-Tween to remove non-bound material. Next, anti-Rabbit IgG (H&L) AF488 (Jackson ImmunoResearch) at a concentration of 5 µg/mL in 3% bovine serum albumin (BSA)-PBS was applied and incubated for 30 minutes to detect bound anti-EGFR antibody or Probody construct. The tissue was then washed in two changes of PBS-T to remove non-bound material and in one wash of PBS. Sections were then counterstained with DAPI and coverslipped using ProLong Gold Anti-Fade Reagent (Invitrogen).

### Image analysis of IHZ assay

Labeled antibodies were visualized using a fluorescence microscope (Olympus IX 81) and Imaging Software for Life Science Microscopy Cell. An image was obtained for each channel. For each label, images were then converted to gray-scale through separation of luminosity using MetaMorph Imaging Software. The average pixel intensity (PI) for each image was determined with the region statistics tool and the values were exported to Microsoft Excel. The ratio of intensity of the Probody molecule staining (Probody PI) or cetuximab staining (antibody PI) in the AF680 or red channel compared to EGFR staining intensity on corresponding antibody (EGFR[Ab]) or Probody construct (EGFR[Pb]) treated tissues in the AF488 or green channel was determined, and the IHZ score (%) calculated as follows:

$${\rm{I}}{\rm{H}}{\rm{Z}}\,{\rm{Score}}\,( \% )=\frac{{\rm{Probody}}\,{\rm{P}}{\rm{I}}\ast {\rm{E}}{\rm{G}}{\rm{F}}{\rm{R}}({\rm{A}}{\rm{b}}){\rm{P}}{\rm{I}}}{{\rm{Antibody}}\,{\rm{P}}{\rm{I}}\ast {\rm{E}}{\rm{G}}{\rm{F}}{\rm{R}}({\rm{P}}{\rm{b}}){\rm{P}}{\rm{I}}}\ast 100$$

### Immunofluorescence staining

Immunofluorescence staining with recombinant antibodies A11 and U33 that recognizes the active form of matriptase and uPA over the zymogen forms, respectively, was performed on frozen tissue sections. Alexa Fluor 750 labeled antibodies were used at a 1:100 dilution. All tissue sections were mounted using ProLong Gold Anti-Fade Reagent containing DAPI (Invitrogen). Imaging was performed using a fluorescence microscope (Olympus IX 81) and Imaging Software for Life Science Microscopy Cell.

### ***In vivo*efficacy studies**

The H292 xenograft model efficacy study was conducted at The Jackson Laboratory and reviewed and approved by the Institutional Animal Care and Use Committee (IACUC). FaDu xenograft model efficacy study was performed at Crown Bioscience and in accordance with the regulations of the Association for Assessment and Accreditation of Laboratory Animal Care (AAALAC).

For the H292 xenograft study conducted at The Jackson Laboratory, 6 to 8 week old female NU/J (JAX #2019) mice were subcutaneously inoculated in the right hind flank with 5 × 10^6^ H292 cells (American Type Culture Collection [ATCC]) suspended 1:1 with Matrigel in serum-free medium. Once tumors become measurable, body weights and tumor volume measurements were made three times weekly. Mice were randomized and grouped in tumor size–matched cohorts (n = 12 mice per group) with an average tumor volume of approximately 150 to 200 mm^3^. Animals were treated intravenously once weekly (25 mg/kg) for 4 weeks. For the FaDu xenograft study performed at Crown Bioscience, 6 to 8 weeks female NOD/SCID mice, weighing approximately 18 to 22 g, were subcutaneously inoculated at the right hind flank with 3 ×10^6^ of FaDu cells in 0.1 mL of PBS. Clinical observations, body weights, and digital caliper tumor volume measurements were made twice weekly once tumors became measurable. Tumor volumes were calculated with the formula (ab^2^)/2, where *a* is the longer and *b* is the shorter of two perpendicular diameters, respectively. When the mean tumor size reached approximately 150 to 200 mm^3^, mice were randomized in cohorts consisting of 8 mice each and were treated intraperitoneally once weekly (25 mg/kg) for 2 weeks.

## Supplememtary information

Supplememtary information.
