# Supplementary material for: Monitoring protease activity in biological tissues using antibody prodrugs as sensing probes
Source: Sci Rep. 2020 Apr 3;10:5894. doi: 10.1038/s41598-020-62339-7 (PMC7125177; doi:10.1038/s41598-020-62339-7)
Supplement: Supplementary file 1 — Supplememtary information. [file 41598_2020_62339_MOESM1_ESM.pdf]

SUPPLEMENTARY INFORMATION

Monitoring protease activity in biological tissues using antibody prodrugs as sensing probes

Olga Vasiljeva[1]*, Elizabeth Menendez[1], Margaret Nguyen[1], Charles S. Craik[2], W. Michael Kavanaugh[1]

Affiliations:

[1]CytomX Therapeutics, Inc.; 151 Oyster Point Blvd. Suite 400, South San Francisco, California, 94080, USA.

[2]Department of Pharmaceutical Chemistry, University of California, 600 16th Street, San Francisco, CA 94143, USA

*e-mail: ovasiljeva@cytomx.com

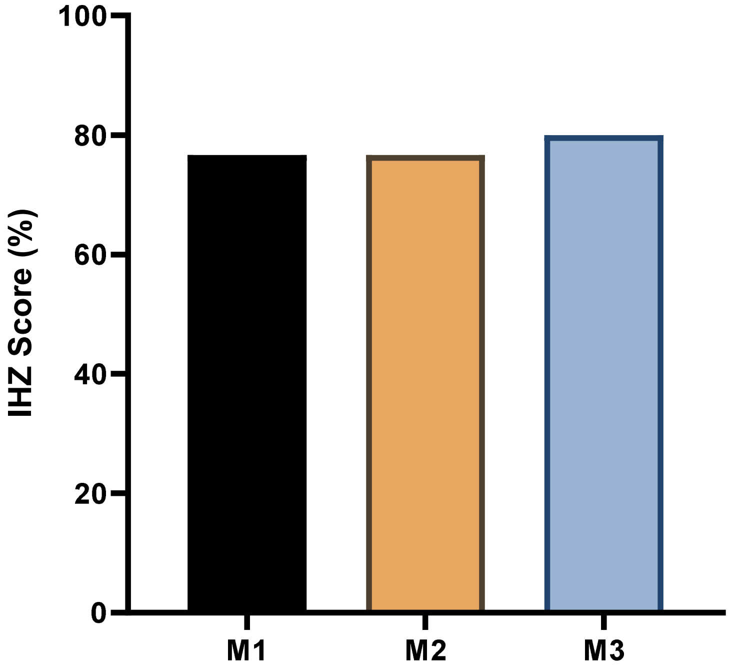

Supplementary Figure 1. Probody-S01 IHZ screening of H292 xenograft tumors obtained from three independent H292 xenograft mice demonstrates similar activation profile between assessed tumors.

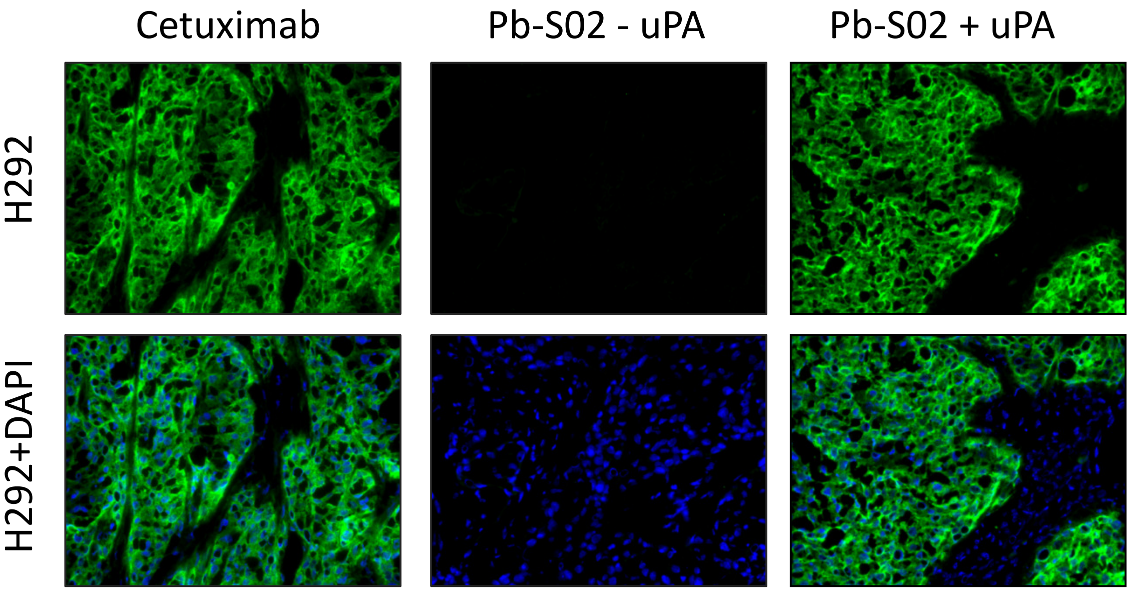

Supplementary Figure 2. Probody-S02 IHZ assay in H292 xenograft tumor tissue in the absence and presence of recombinant uPA (staining in green). DAPI nuclear staining appears in blue.

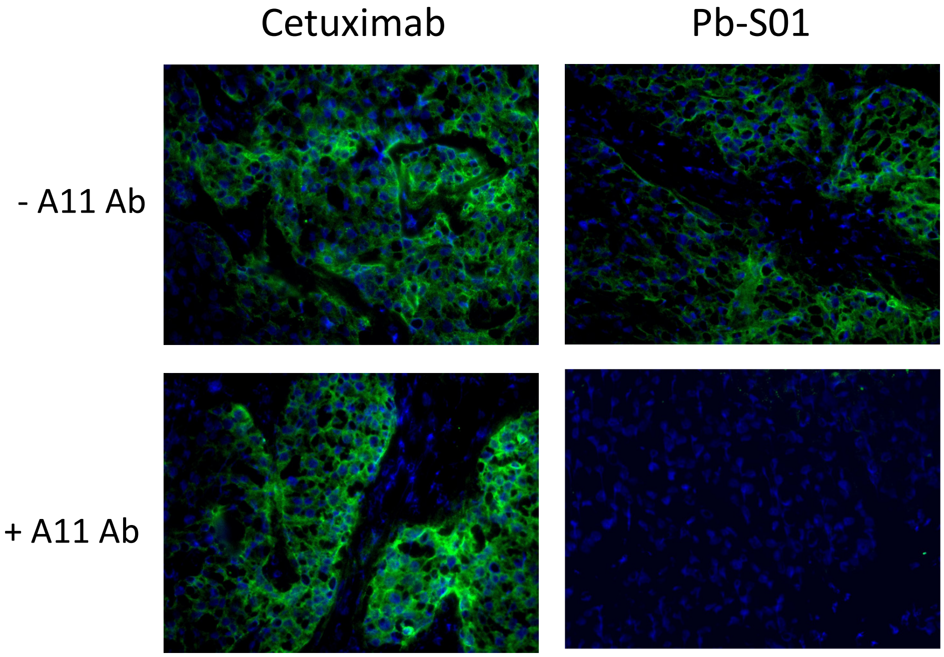

Supplementary Figure 3. Inhibition of Probody-S01 IHZ signal (staining in green) by pre-treatment of H292 xenograft tissue with A11 active-site matriptase inhibitory antibody. DAPI nuclear staining appears in blue.

Supplementary Table 1. EGFR IHC and Probody-S01 IHZ screening of CRC patient-derived xenograft (PDX) tumor models

PDX model	EGFR IHC	Pb-S01 IHZ (%)
CRC_PDX-1	++	100
CRC_PDX-2	+++	90
CRC_PDX-3	+++	65
CRC_PDX-4	+++	90
CRC_PDX-5	+++	90
CRC_PDX-6	+++	80
CRC_PDX-7	+++	70
CRC_PDX-8	++	85
CRC_PDX-9	++	90
CRC_PDX-10	++	90
CRC_PDX-11	+++	100
CRC_PDX-12	+++	90
CRC_PDX-13	++	95
CRC_PDX-14	++	90
CRC_PDX-15	++	75

Supplementary Table 2. Summary of Probody-S02 and Probody-M02 IHZ screening of TNBC patient samples

Patient #	Stage	Her-2 neu	ER	PR	Cetuximab	Pb-S02, %	Pb-M02, %
#1	IIIA	0	-	-	+	60	40
#2	IIIB	0	-	-	+	40	50
#3	IIA	0	-	-	++	100	95
#4	IIA	0	-	-	+++	10	85
#5	IIIA	0	-	-	++	65	100
#6	IIA	1+	-	-	++	85	100
#7	IIA	0	-	-	++	25	85
#8	IIA	1+	-	-	+	25	100
#9	IIA	0	-	-	++	60	30
